# Control in asthma and COPD – correct inhaler technique is crucial

**DOI:** 10.7196/AJTCCM.2019.v25i3.028

**Published:** 2019-09-17

**Authors:** E M Irusen

**Affiliations:** Division of Pulmonology, Faculty of Medicine and Health Sciences, Stellenbosch University, Cape Town, South Africa

## Editorial

‘The longest part of my consultation deals with inhaler technique’ is the surprise response that the audience receives during a lecture, when I am asked about my approach to the difficult asthmatic. The issue of the ‘difficult asthmatic’ is a frequent problem in clinical practice. The two commonest reasons for uncontrolled asthma are non-adherence to prescriptions and an inadequate inhaler technique.^[1,2]^ Having ascertained the former, it then behoves the healthcare practitioner to check and reinforce the inhaler technique. In my personal experience, I have seen patients who have never been taught how to use an inhaler, nor has their inhaler technique ever been checked in up to 20 years of having had asthma.

The vast majority of asthmatics are managed with inhaled therapy alone; this usually takes the form of a metered-dose inhaler (MDI). Frequently the MDI is prescribed, dispensed by the pharmacist, and it is usually presumed that the subject will self-learn; but failure to master the technique is virtually guaranteed. Exacerbating this factor is the availability to patients of a bewildering variety of inhalers, both pressurised and dry-powder. The assortment of inhalers can be a challenge to practitioners as well. All facilities and practices should ensure that personnel trained in the correct use of inhalers are available to assist patients. There are several focussed journals on aerosol medications, so the topic can be quite intricate; those regularly involved in the care of respiratory patients are encouraged to access some of these articles.^[3–5]^

There are several steps in performing an adequate inhalation from an MDI. It also comes as a surprise to practitioners when they learn that only 10 - 25% of a dispensed dose reaches the lower respiratory tract. Therefore, if subjects do not take a full inspiration after a complete exhalation or there is no breath-holding after breathing in the medication, negligible amounts will have actually been delivered to the lung; hence the poor therapeutic effect. These are some of the deficits in manoeuvres that are considered critical errors. All too often, in this type of situation, more potent or additional inhalers are prescribed, adding substantially to costs, but with little clinical improvement.^[6,7]^ The problem may be further compounded when a patient uses different types of inhaler, aggravating the errors of each device; a move towards single inhaler platforms may mitigate this issue in the future.

The problems of inadequate performance in the use of inhalers have been recognised since their inception^[8]^ and there have been regular reports of such ongoing problems;^[9]^ the article in the current issue of this journal therefore comes as no surprise. Both medical students and qualified personnel exhibited errors in the use of MDIs. Whilst education and reinforcement of inhaler technique are important, the persistence of these issues over decades suggests that changes in strategy are needed. As technology advances and becomes more accessible, this resource could be better harnessed. Patients could download apps that serve as reminders for adherence or access to videos that demonstrate correct technique. Devices are also becoming more sophisticated; for example, Propeller Health have developed a sensor that clicks onto the inhaler and links the data to a smartphone that provides personalised insights into patients’ disease control.^[10]^

In conclusion, addressing and reinforcing inhalation technique is the most overlooked aspect of management in the obstructive lung diseases, and has major personal and public health ramifications. There is still an obligation to ensure patients are adequately educated and trained on devices. Hopefully, in the future, as digital technology better complements our efforts, the situation will improve.

## References

[1] Bourdin A, Halimi L, Vachier I (2012). Adherence in severe asthma.. Clin Exp Allergy.

[2] Khurana AK, Dubey K, Goyal A, Pawar KS, Phulwaria C, Pakhare A (2019). Correcting inhaler technique decreases severity of obstruction and improves quality of life among patients with obstructive airway disease.. J Family Med Prim Care.

[3] Rottier BL, Rubin BK (2013). Asthma medication delivery: Mists and myths.. Paediatr Respir Rev.

[4] Smaldone GC, Berg E, Nikander K (2005). Variation in pediatric aerosol delivery: Importance of facemask.. J Aerosol Med.

[5] Pitcairn GR, Lim J, Hollingworth A, Newman SP (1997). Scintigraphic assessment of drug delivery from the Ultrahaler dry powder inhaler.. J Aerosol Med.

[6] Kocks JWH, Chrystyn H, van der Palen J (2018). Systematic review of association between critical errors in inhalation andhealth outcomes in asthma and COPD.. NPJ Prim Care Respir Med.

[7] Lewis A, Torvinen S, Dekhuijzen PN (2016). The economic burden of asthma and chronic obstructive pulmonary disease and the impact of poor inhalation technique with commonly prescribed dry-powder inhalers in three European countries.. BMC Health Serv Res.

[8] Hanania NA, Wittman R, Kesten S, Chapman KR (1994). Medical personnel’s knowledge of and ability to use inhaling devices. Metered-dose inhalers, spacing chambers, and breath-actuated dry-powder inhalers.. Chest.

[9] Cho-Reyes S, Celli BR, Dembek C, Yeh K, Navaie M (2019). Inhalation technique errors with metered-dose inhalers among patients with obstructive lung diseases: A systematic review and meta-analysis of U.S. studies.. Chronic Obstr Pulm Dis.

[10] Merchant R, Szefler SJ, Bender BG (2018). Impact of a digital health intervention on asthma resource utilization.. World Allergy Organ J.

